# Network Mendelian randomization: using genetic variants as instrumental variables to investigate mediation in causal pathways

**DOI:** 10.1093/ije/dyu176

**Published:** 2014-08-22

**Authors:** Stephen Burgess, Rhian M Daniel, Adam S Butterworth, Simon G Thompson, the EPIC-InterAct Consortium

**Affiliations:** ^1^Department of Public Health and Primary Care, University of Cambridge, Cambridge, UK and ^2^Department of Medical Statistics, London School of Hygiene and Tropical Medicine, London, UK

**Keywords:** Mendelian randomization, mediation, instrumental variable, direct effect, indirect effect

## Abstract

**Background:** Mendelian randomization uses genetic variants, assumed to be instrumental variables for a particular exposure, to estimate the causal effect of that exposure on an outcome. If the instrumental variable criteria are satisfied, the resulting estimator is consistent even in the presence of unmeasured confounding and reverse causation.

**Methods:** We extend the Mendelian randomization paradigm to investigate more complex networks of relationships between variables, in particular where some of the effect of an exposure on the outcome may operate through an intermediate variable (a mediator). If instrumental variables for the exposure and mediator are available, direct and indirect effects of the exposure on the outcome can be estimated, for example using either a regression-based method or structural equation models. The direction of effect between the exposure and a possible mediator can also be assessed. Methods are illustrated in an applied example considering causal relationships between body mass index, C-reactive protein and uric acid.

**Results:** These estimators are consistent in the presence of unmeasured confounding if, in addition to the instrumental variable assumptions, the effects of both the exposure on the mediator and the mediator on the outcome are homogeneous across individuals and linear without interactions. Nevertheless, a simulation study demonstrates that even considerable heterogeneity in these effects does not lead to bias in the estimates.

**Conclusions:** These methods can be used to estimate direct and indirect causal effects in a mediation setting, and have potential for the investigation of more complex networks between multiple interrelated exposures and disease outcomes.

**Key Messages**

- When instrumental variables are available for an exposure and mediator in a causal network, the direct and indirect effects of the exposure on an outcome, controlling for the mediator, can be estimated in the presence of unmeasured confounding in the model considered. The direction of causal effect between the exposure and mediator can also be verified.
- Formally, strong assumptions of linearity without interaction and homogeneity of causal effects are required for the consistency of estimators, although simulation analyses suggest that estimates may be robust to substantial random heterogeneity.
- The methods presented have potential application in the context of Mendelian randomization for the estimation of causal networks.

## Introduction

The technique of Mendelian randomization is being extensively applied to estimate the long-term causal effects of various exposures on clinical and epidemiological outcomes using observational data. It employs genetic variants to remove bias due to confounding and reverse causation.^1^ These variants must satisfy the assumptions of an instrumental variable (IV): association with the exposure of interest; lack of association with any confounder of the exposure–outcome relationship (including those that are unmeasured); and lack of conditional association with the outcome given the exposure and all the confounders.^2^ Such a genetic variant divides the observed population into subgroups which differ systematically with respect to the exposure of interest and any causal descendants thereof, but not with respect to potential confounding variables.^3^ These subgroups are analogous to arms in a randomized controlled trial where the intervention is to change the level of the exposure.^4^

**Figure 1.**
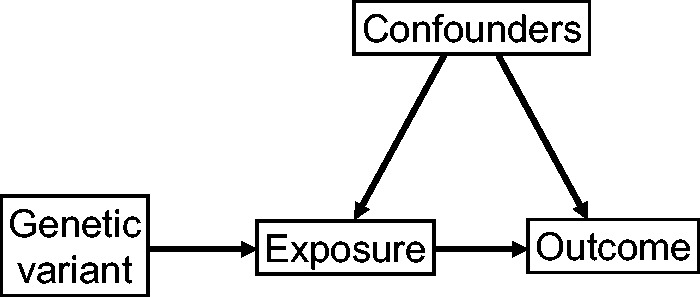
Causal directed acyclic graph (DAG) of Mendelian randomization assumptions.

The usual scenario investigated in Mendelian randomization is given in the causal directed acyclic graph (DAG) of Figure 1, which illustrates the assumed relations between the genetic variant, exposure, outcome and confounders.^5^ The observational correlation between the exposure and the outcome does not have a causal interpretation, due to the presence of confounding variables, which may be unobserved. The IV assumptions about the relationship of the genetic variant with the other variables enable identification and consistent estimation of the causal effect of the exposure on the outcome.^6^ In fact, unlike Figure 1, a genetic variant used in a Mendelian randomization analysis need not necessarily be causally related to the exposure; it may be a proxy for the true causal variant. Any variant in linkage disequilibrium (meaning correlated in its distribution) with the causal variant which satisfies the IV assumptions can be used as an IV.7^7^

As genetic research progresses, the number of risk factor variables (exposures, biomarkers or other potential risk factors) with associated genetic variants, where there is enough biological knowledge to use the variants as IVs, is rapidly increasing.^8^ If multiple risk factors with associated IVs have been measured in the same dataset, then the causal effect of each of the risk factors on the outcome can be estimated. Additionally, each of the risk factors can be considered as the outcome in an IV analysis, and the causal effects of the risk factors on each other can be estimated. Such estimates could give insight into the causal network of relations between multiple risk factors, which is informative about the mechanisms between them and the outcome.^9^ This has been proposed in the context of integrating data on genetic variants with ‘omics’ data, such as data on gene expression, epigenetic markers and metabolites.^10^ Potential areas of application of this technique in high-throughput datasets are considered in the discussion.

In this work, we seek to extend conventional Mendelian randomization analysis by considering a simple causal network of risk factors. We suppose that the causal effect of an exposure on an outcome is partially mediated by another risk factor. The total effect of the exposure on the outcome can therefore be decomposed into a direct and an indirect effect.^11^^,^^12^ The direct effect of the exposure is the effect on the outcome of manipulating the exposure while the mediator remains unchanged. If this is achieved by fixing the mediator at a given value for all individuals, then this is a controlled direct effect. If it is achieved by letting the mediator take the value it would have taken if the exposure were unchanged (which will differ between individuals), then this is a natural direct effect. The natural indirect effect is the residual effect on the outcome of the change in the mediator attributable to a change in the exposure.^13^ Formal definitions of these quantities require the mathematical language of counterfactuals, and are given in the Web Appendix (available as Supplementary data at *IJE* online). To give a motivating example, the causal effect of smoking on coronary heart disease risk may be partially mediated by the effect of smoking on blood pressure. If the mediation is substantial and the direct effect of smoking on heart disease is small compared with the total effect, then an intervention on blood pressure may be as effective to reduce heart disease risk among smokers as an intervention on smoking itself. We note that the term ‘direct effect’ depends on the choice of the mediator, as the effect includes pathways which are not direct in any absolute sense, but are mediated by variables other than the mediator under consideration.

The structure of this paper is as follows. We first discuss two methods to estimate direct and indirect effects using genetic variants as IVs for the exposure and the mediator. The methods are initially presented informally, followed by a technical discussion of the parameters estimated and the necessary assumptions. We then illustrate the application of the methods in a simulation study and in an applied example, paying particular attention to the impact on effect estimates of violations of parametric assumptions (such as linearity and constant effects across individuals), and conclude with a discussion of the limitations of the methods and their future potential.

## Methods

**Figure 2.**
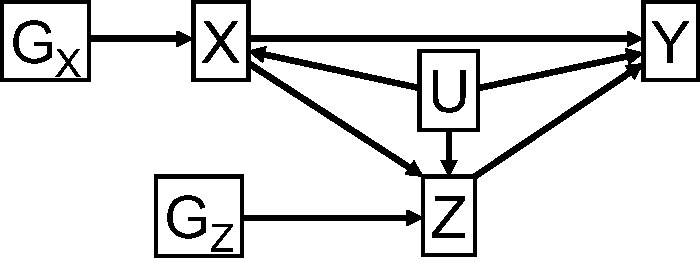
Causal directed acyclic graph (DAG) leading to direct and indirect causal effects of variable *X* on *Y* with mediator *Z*, associated instrumental variables *G_X_* and *G_Z_*, and confounders *U*.

We consider the causal effect of an exposure *X* on an outcome *Y* with a mediator *Z*. The exposure and mediator each have corresponding genetic IVs, *G_X_* and *G_Z_* respectively. A causal DAG illustrating the relationships between these variables is given in Figure 2. We consider the situation where the exposure, mediator and outcome are all continuous and assume that the effects of the exposure on the mediator (*X* on *Z*), and of the exposure and mediator on the outcome [(*X*, *Z*) on *Y*] are linear without interactions. Similar methods could be used in a case of a binary exposure, mediator and/or outcome, but we do not address the additional complications of non-collapsibility that arise in this paper.^14^^,^^15^ We allow unmeasured confounding of the exposure–mediator, exposure–outcome and mediator–outcome relationships. This is indicated by a single variable *U* on the DAG; however, this can be thought of as a vector containing several components corresponding to different confounders, some of which may not be associated with all of *X*, *Z* and *Y*. For simplicity of presentation, the DAG in Figure 2 does not include ‘post-treatment confounders’.^16^ These are confounders of the relationship between the mediator and outcome which are affected by changes in the exposure, and are discussed in the next section.

We discuss two methods for the estimation of direct and indirect effects: a regression-based method, which can be understood by those already familiar with standard IV methods for Mendelian randomization as a repeated application of the ratio method or as an extension of the two-stage least squares method; and a structural equation modelling approach, which is more easily generalizable to more complex causal networks.

### Regression-based methods

The ratio method (or Wald method) is a simple method for estimating a total causal effect with a single IV. The coefficient from the regression of the outcome on the exposure’s IV (${\hat{\beta }}_{Y|G_{X}}$) is divided by the coefficient from the regression of the exposure on the IV (${\hat{\beta }}_{X|G_{X}}$):

(1) $${\hat{\beta }}_{X\to Y}=\frac{{\hat{\beta }}_{Y|G_{X}}}{{\hat{\beta }}_{X|G_{X}}}$$

where → represents a total causal effect.^17^ The same estimate can be obtained using the two-stage least squares (2SLS) method, by regressing the exposure on the IV to obtain fitted values of the exposure ($\hat{X}|G_{X}$), and then regressing the outcome on these fitted values.^18^ The 2SLS method can also be used with multiple IVs. The ratio estimate has been named the linear IV average effect as it represents the causal effect of the exposure on the outcome averaged across the population on a linear scale.^3^

If all effects are linear without interaction terms, the natural direct effect of *X* on *Y* not mediated by *Z* can be obtained, under the assumption of homogeneity of causal effects across individuals in the population, as the difference between the total effect of *X* on *Y* and the product of the effects of *X* on *Z* and *Z* on *Y.*^19^ The causal effects of *X* on *Z* and of *Z* on *Y* can each be estimated by application of the ratio method, so the natural direct causal effect is:

(2) $$\begin{array}{c} {\hat{\beta }}_{X\Rightarrow Y}={\hat{\beta }}_{X\to Y}-{\hat{\beta }}_{X\to Z}{\hat{\beta }}_{Z\to Y}\\ =\frac{{\hat{\beta }}_{Y|G_{X}}}{{\hat{\beta }}_{X|G_{X}}}-\;\frac{{\hat{\beta }}_{Z|G_{X}}}{{\hat{\beta }}_{X|G_{X}}}\frac{{\hat{\beta }}_{Y|G_{Z}}}{{\hat{\beta }}_{Z|G_{Z}}} \end{array}$$

where ⇒ represents a natural direct effect. The natural indirect effect is ${\hat{\beta }}_{X\to Z}{\hat{\beta }}_{Z\to Y}$. The standard error and confidence intervals for these quantities can be estimated by bootstrapping. If the natural direct effect is constant with respect to the mediator for all individuals, then it is equal to the controlled direct effect for all values of the mediator.^13^ We therefore henceforth omit the reference to natural or controlled direct effects in the context of linear models without interactions unless we are specifically differentiating between the two.

**Figure 3.**
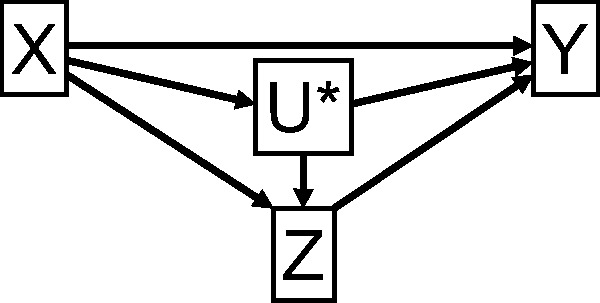
Causal directed acyclic graph (DAG) illustrating direct and indirect causal effects of variable *X* on *Y* with mediator *Z* with post-treatment confounder U*.

If there are post-treatment confounders U* (Figure 3), then the natural direct effect cannot in general be identified (even if these variables are measured^20^) without further assumptions.^21^ Maintaining the assumption that effects are linear without interaction terms, the total causal effect of *X* on *Y* can be further decomposed into:
(3)
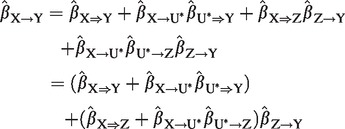

which is simply the sum of the direct and indirect effects as before. Therefore, we can omit specific reference to post-treatment covariates in the context of linear models without interactions.

Similar estimates of the direct and indirect effects corresponding to equation (2) can also be obtained by a ‘multiple-stage least squares’ approach, where the first stage is to obtain fitted values of the exposure on its IV ($\hat{X}|G_{X}$), then to obtain fitted values of the mediator regressed on its IV and the fitted values of the exposure [ ($\hat{Z}|(G_{Z},\hat{X}|G_{X})$)], and the final stage is to regress the outcome on the fitted values $\hat{X}|G_{X}$ and $\hat{Z}|(G_{Z},\hat{X}|G_{X})$. This approach is discussed by Tchetgen Tchetgen and Lin,^22^ who give a justification of the method starting from the non-parametric structural equation modelling framework of Pearl.^23^

### Structural equation models

**Figure 4.**
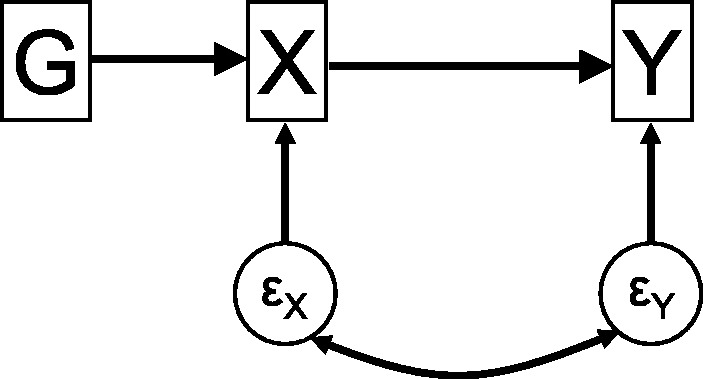
Path diagram for estimation of causal effect of exposure (*X*) on outcome (*Y*) in the presence of unmeasured confounding using instrumental variable (*G*).

Alternatively, parameters in this causal network and other more complex networks can be estimated using structural equation models (SEMs). SEMs are used extensively in the social sciences for inference on the network of associations between variables.^24^ A SEM is a compound hypothesis about the relations between measured and latent variables as encoded in a path diagram. Assuming that the path diagram is correctly specified, coefficients from a SEM can be viewed as representing causal effects, although the causal nature of the estimates is by prior assumption rather than being empirically established by the data.^25^ IV analysis can be performed in a SEM framework as the IV assumptions can be used to define a causal path diagram under which the data can be analysed. Relations between variables can be represented by directed arrows, indicating a causal effect, often assumed to be linear, or bidirectional arrows, indicating a correlation between variables. Measured variables are represented by squares, and latent variables, including measurement error terms, by circles. In the IV path diagram (Figure 4) corresponding to the DAG in Figure 1, the unmeasured confounding between the exposure (*X*) and the outcome (*Y*) is modelled by allowing correlation in the path diagram between their respective error terms $\epsilon_{X}$ and $\epsilon_{Y}$.

**Figure 5.**
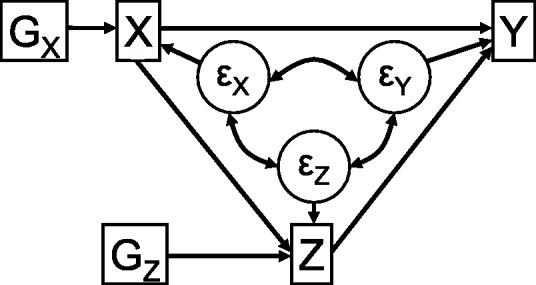
Path diagram for estimation of causal direct and indirect effects of exposure (*X*) on outcome (*Y*) with mediator (*Z*) in the presence of unmeasured confounding using instrumental variables (*G_X_,G_Z_*) in a structural equation model (SEM) framework.

To estimate direct and indirect effects in a SEM framework, we assume a path diagram corresponding to Figure 5. This is similar to Figure 2 except that the unmeasured confounding is expressed as a correlation between the error terms $\epsilon_{X}$, $\epsilon_{Z}$ and $\epsilon_{Y}$. The model is identified by the directional assumptions about the effects of the IVs on the variables which they instrument. The coefficients in the SEM represent the direct effects between individual variables. The indirect effect of *X* on *Y* via *Z* can be calculated under the assumptions of linearity and homogeneity of effects without interactions either by estimating the total effect of *X* on *Y* assuming the path diagram of Figure 4 and subtracting the direct effect, or (as in this paper) by multiplying the coefficient for the causal effect of *X* on *Z* by that for the causal effect of *Z* on *Y*.

In this work, we use the *sem* function in Stata 12^26^ for SEM analyses, estimating parameters by maximum likelihood. In the conventional IV setting (Figure 1), this is also known as full information maximum likelihood (FIML).^27^ Confidence intervals can be constructed based on asymptotic standard errors. A standard error for the indirect effect can be calculated from the delta method in Stata using the *nlcom* function. A useful feature of SEMs is the availability of tests for assessing goodness-of-fit of the model.^28^ Estimation of parameters and many goodness-of-fit tests rely on the assumption of multivariate normality of the variables.^29^

### Direction of the causal effect

In the set-up of Figure 2, but where it is uncertain whether *Z* is a mediator of *X* or vice versa, it is additionally possible to test for a causal effect between *X* and *Z* in both directions. IVs for *X* can be used to estimate the causal effect of *X* on *Z*, and IVs for *Z* can be used to estimate the causal effect of *Z* on *X*. These estimates can be used to orientate the direction of causal effect (if any) between the exposure and mediator. Such an analysis has been named ‘reciprocal Mendelian randomization’.^30^

As genetic subgroups of a population defined by an IV represent subpopulations with long-term average differences in the exposure of interest,^31^ the causal effects estimated in a Mendelian randomization analysis represent long-term relationships, equivalent to a randomized trial where the intervention is made at conception. As such, changes in the effects of the exposure and mediator over time and feedback between the exposure and mediator cannot be addressed by a conventional Mendelian randomization analysis. This has consequences for the interpretation of all Mendelian randomization estimates,^32^ and particularly in a mediation setting, where a ‘bidirectional’ causal relationship between *X* and *Z* may reflect an effect of (say) *X* on *Z* in early life, and *Z* on *X* in later life. Ideally in mediation analyses, biological knowledge should be used to provide a causal ordering of the exposure, mediator and disease. Where this is not possible, reciprocal Mendelian randomization approaches may provide evidence on the direction of causal effects, although all such estimates rely on the assumption that these effects do not vary in direction over time.

### Technical issues

Although the concepts of a direct and indirect effect can be understood intuitively, precise definitions depend on exactly how the interventions on the exposure and mediator are performed.^11^^,^^13^ A controlled direct effect is the effect of increasing the exposure when the mediator is set to be fixed at a given level. A natural direct effect is the effect of increasing the exposure when the mediator is left at the level it would have taken had the exposure been observed at its reference value. The controlled direct effect is an appealing quantity as it can be estimated as the result of an experiment when the levels of the exposure and mediator can be separately manipulated. The natural direct effect requires an estimate of the outcome as if the exposure were intervened on, but the mediator took its value as if the exposure took a different value. This is intrinsically a counterfactual quantity, and as such cannot be observed from any experiment.^33^ However, the natural direct effect has a counterpart natural indirect effect: the effect of increasing the mediator from the level it would take if the exposure took its reference value to the level it would take if the exposure were increased, keeping the exposure at its elevated level. The total effect of the exposure on the outcome is equal to the sum of the natural direct and indirect effects.^11^^,^^13^ These definitions are discussed further in the Web Appendix (available as Supplementary data at *IJE* online).

The method of IVs exploits a natural experiment, enabled by the random distribution of the IV in the population. The IV acts to change the variable which it instruments. In the context of mediation, the use of separate IVs for *X* and for *Z* can be viewed as separate experiments to set the values of *X* and *Z*,^34^ and so using IVs in a non-parametric setting to estimate the distributions of the exposure and mediator would allow the calculation of a controlled direct effect. However this is equal to the natural direct effect in the linear setting if the controlled direct effect is constant for all values of the mediator, that is if there is no interaction between *X* and *Z* in their effect on *Y*.^13^ In contrast, the analogous parallel design approach of Imai *et al*., in which two experiments are performed to affect the values of the exposure and mediator separately, is proposed to target a natural direct effect parameter (although different views were expressed as to the appropriate target parameter in the commentary on the paper).^34^ However the authors make the same no-interaction assumption as stated above, rendering this discussion to a large degree a question of philosophical preference rather than one having any applied consequence. In the context of Mendelian randomization, where exposure and mediator variables are usually continuous, the assumption of linear effects is often made to allow the presentation of a single effect estimate for all levels of the variable of interest.

The reliance on separate experiments and the decomposition of the indirect effect into the product of separate effects on the mediator and outcome can lead to incorrect inferences if the causal effects of *X* on *Z* and of *Z* on *Y* vary substantially for different individuals in the population. This is known as the fallacy of the causal chain approach^35^ It is even possible for the average causal effects of the exposure on the mediator and of the mediator on the outcome to be positive, but for the average indirect effect to be negative. This is an analogous problem to Simpson’s paradox, whereby the average effect in the population can be in the opposite direction to the average effects in each of the substrata of the population.^36^^,^^37^ Hence the use of IVs for both the exposure and the mediator formally requires the assumption of homogeneity across individuals of the effects of a unit change in the exposure on the mediator and on the outcome, as well as of a unit change in the mediator on the outcome. As linearity is assumed here, these effects are also required to be constant for all values of the exposure and mediator.

## Simulations

We now perform a simulation study to demonstrate the use of the two methods discussed above, regression-based and SEM, to provide estimates of direct and indirect effects. We also use these simulations to assess the impact of heterogeneity of causal effects on these estimates. Data were simulated on 5000 individuals indexed by *i* from the following plausibly realistic data-generating model, corresponding to Figures 2 and 5:
(4)
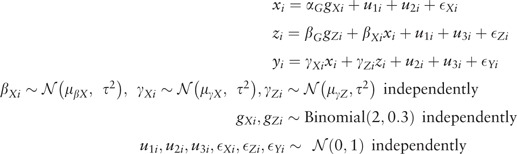



The IVs *G_X_* and *G_Z_* are modelled to correspond to biallelic genetic variants (taking values 0, 1, 2) with a minor allele frequency of 0.3. The *U* variables represent confounders in the associations between the exposure, mediator and outcome. *X* and *Z* take both positive and negative values.

The causal effects are allowed to vary between individuals; for example the causal effect of *X* on *Z* ($\beta_{Xi}$) has mean $\mu_{\beta X}$ and standard deviation *τ*; *τ* = 0 corresponds to no variability between individuals (homogeneity), and *τ* > 0 to variability (heterogeneity). We set $\mu_{\gamma X}$ = 1 throughout, so that the average direct effect is 1 in all scenarios, and take $\mu_{\beta X}$, $\mu_{\gamma Z}$ = ±1. The average indirect effect is $\mu_{\beta X}\mu_{\gamma Z}$. Three values of *τ*^2^ are considered: 0, 0.2^2^ and 0.4^2^, representing respectively no, moderate and substantial heterogeneity. So 12 scenarios are considered in total. We set *α_G_* = 0.3, and $\beta_{G}$ = 0.5, when $\mu_{\beta X}$ = 1 and $\beta_{G}$ = 0.36 when $\mu_{\beta X}$ = −1, so that the averageproportion of the variance in *X* and *Z* explained by the IVs *G_X_* and *G_Z_*_,_ respectively, (the coefficient of determination, R^2^) is 1.3% in all scenarios. With a sample size of 5000, this corresponds to average F statistics of around 65, so the potential of bias from weak instruments is small.^38^

The impact of interaction between *X* and *Z* in the generating model for *Y* is considered in the Web Appendix (available as Supplementary data at *IJE* online): first by adding an interaction term with zero mean but non-zero variance, so that there is interaction between *X* and *Z* on an individual level, but not on average; and then by adding an interaction term with non-zero mean. Additionally, the impacts of heterogeneity in the genetic effects of *G_X_* and *G_Z_* on *X* and *Z*, respectively, and of correlation in the causal effects between *X*, *Z* and *Y*, are considered.

## Results

For each set of parameter values, 1000 datasets were generated. The causal effects of *X* on *Z* and of *Z* on *X* were estimated using the ratio method, and the direct and indirect effects of *X* on *Y* for the mediator *Z* were estimated using regression-based and SEM methods. Out of the 12 000 datasets, using a 5% significance level, a causal effect of *X* on *Z* was found in all but seven datasets (>99.9%). A causal effect of *Z* on *X* was found in 4.8% of datasets, which is no more than would be expected by chance alone. Mean estimates of the direct and indirect effects across simulations are given in Table 1, as well as the mean standard error of estimates (in the regression-based analyses, standard errors were calculated by bootstrapping with 1000 bootstrap resamples; in the SEM analyses, they were calculated analytically), and the standard deviation of estimates. The Monte Carlo standard error of the mean estimates, representing the uncertainty due to the finite number of datasets, is around 0.005.

**Table 1.** Mean estimates, mean standard errors (SE) and standard deviations of estimates (SD) of the direct and indirect effects of *X* on *Y* controlling for *Z*; from regression-based and structural equation model (SEM) methods in simulation study: $\mu_{\gamma X\;}$ = average direct effect of *X* on *Y*, $\mu_{\beta X}$ = average effect of *X* on *Z*, $\mu_{\gamma Z}$ = average effect of *Z* on *Y*, $\mu_{\beta X}\mu_{\gamma Z\;}$ = average indirect effect of *X* on *Y* mediated by *Z*, *τ* = heterogeneity in individual-level causal effect parameters

Regression-based										
Direct effect ($\mu_{\mathrm{\gamma X}}=1$)		τ^2 ^= 0			τ^2 ^= 0.2^2^			τ^2 ^= 0.4^2^		

$\mu_{\mathrm{\beta X}}$	$\mu_{\mathrm{\gamma Z}}$	Mean	SE	SD	Mean	SE	SD	Mean	SE	SD
1	1	1.00	0.19	0.19	1.01	0.20	0.19	1.00	0.23	0.22
1	−1	1.01	0.20	0.18	1.00	0.20	0.19	1.00	0.23	0.23
−1	1	1.00	0.22	0.21	1.01	0.23	0.22	1.00	0.26	0.25
−1	−1	1.01	0.23	0.21	1.00	0.24	0.22	1.00	0.26	0.24

Indirect effect ($\mu_{\mathrm{\beta X}}\mu_{\mathrm{\gamma Z}}$)		τ^2^^ ^= 0			τ^2^^ ^= 0.2^2^			τ^2^^ ^= 0.4^2^		

1	1	0.98	0.20	0.19	0.98	0.20	0.19	0.98	0.21	0.20
1	−1	−1.01	0.20	0.19	−1.01	0.20	0.19	−1.01	0.21	0.21
−1	1	−1.02	0.22	0.22	−1.01	0.23	0.22	−1.02	0.25	0.24
−1	−1	0.99	0.23	0.21	1.00	0.23	0.23	1.00	0.25	0.24

Structural equation models (SEM)										

Direct effect ($\mu_{\mathrm{\gamma X}}=1$)		τ^2^^ ^= 0			τ^2^^ ^= 0.2^2^			τ^2^^ ^= 0.4^2^		

$\mu_{\mathrm{\beta X}}$	$\mu_{\mathrm{\gamma Z}}$	Mean	SE	SD	Mean	SE	SD	Mean	SE	SD
1	1	0.99	0.15	0.15	0.99	0.16	0.16	0.99	0.19	0.19
1	−1	1.00	0.15	0.15	0.99	0.16	0.16	0.99	0.19	0.19
−1	1	1.00	0.16	0.16	1.00	0.17	0.17	0.99	0.20	0.20
−1	−1	0.99	0.17	0.17	0.99	0.17	0.17	0.99	0.20	0.20

Indirect effect ($\mu_{\mathrm{\beta X}}\mu_{\mathrm{\gamma Z}}$)		τ^2^^ ^= 0			τ^2^^ ^= 0.2^2^			τ^2^^ ^= 0.4^2^		

1	1	0.99	0.15	0.15	1.00	0.15	0.16	0.99	0.17	0.17
1	−1	−1.00	0.15	0.15	−1.00	0.15	0.15	−1.01	0.17	0.17
−1	1	−1.01	0.16	0.16	−1.00	0.17	0.17	−1.01	0.19	0.19
−1	−1	1.01	0.17	0.17	1.01	0.17	0.17	1.01	0.19	0.19

Both methods appear to estimate the average direct and indirect effects without substantial bias, even when there is individual-level heterogeneity of effects (τ^2^^ ^> 0). The mean standard errors agreed well with the empirical standard deviations of the estimates. Estimates using the SEM method seemed to be more efficient, with estimates having lower mean standard errors than those from the regression-based method. This corresponds to the stronger distributional assumption of multivariate normality, which is satisfied in this example, made by the SEM method. A similar finding of no substantial bias was found when there was a zero mean interaction between *X* and *Z* in the model for *Y* (Web Table A1, available as Supplementary data at *IJE* online), although some bias was observed when the interaction term had non-zero mean (Web Table A2, available as Supplementary data at *IJE* online). Under heterogeneity in the genetic effects on the exposure and mediator, results were not materially different to those in the original simulation (Web Table A3, available as Supplementary data at *IJE* online). Under correlation in the causal effect parameters $\beta_{Xi}$, $\gamma_{Xi}$, $\gamma_{Zi}$, there was a slight bias in estimates of the direct effect in the direction of the correlation when there was substantial heterogeneity in the parameters, but no evident bias in estimates of the indirect effect (Web Table A4, available as Supplementary data at *IJE* online).

We conclude that estimates of the direct and indirect effects using the methods presented in this paper are robust to quite substantial random heterogeneity in the causal and genetic effects, and to random (zero mean) individual-level interaction, in the range of simulation examples considered.

## Example: body mass index, C-reactive protein and uric acid

To illustrate these approaches, we consider the causal relationships between body mass index (BMI, kg/m^2^), C-reactive protein (CRP, mg/l) and uric acid (mg/dl). Previous research has shown that genetic variants associated with BMI are associated with CRP levels^30^^,^^39^ and associated with uric acid concentrations,^40^ although in both cases the reverse was not found for genetic variants which are plausible IVs for CRP and for uric acid. We verify the directions of causal effects between these variables, and additionally consider the direct and indirect causal effects of BMI on uric acid using CRP as a potential mediator. Data were taken on 7158 subcohort participants from 20 centres of European ancestry from the EPIC-InterAct study,^41^ a multicentre case-cohort study of type 2 diabetes nested within the European Prospective Investigation into Cancer and Nutrition (EPIC) with complete data on the three variables (BMI, CRP, uric acid). To simplify the analysis, a weighted allele score was constructed out of the genetic variants for each of the variables.^42^ Details of the genetic variants and the construction of the allele scores are given in the Web Appendix (available as Supplementary Data at *IJE* online). CRP was log-transformed throughout.

The coefficients, standard errors, and *P*-values from the regressions of each of the variables on an allele score for each of the other variables are given in Table 2. In each regression, adjustment is made for age, sex and centre. Allele scores are scaled so that the coefficient of each allele score in the regression on the variable it instruments is 1. The allele score for BMI is associated with CRP (*P* = 0.009), whereas the allele score for CRP is not clearly associated with BMI (*P* = 0.17), suggesting that increases in BMI cause increases in CRP levels, but the opposite is not true. The allele score for BMI is not associated with uric acid (*P* = 0.12), although the direction of the association is consistent with that previously observed.^40^ The allele score for uric acid is not associated with BMI (*P* = 0.57). Using equation (2), the direct effect of BMI on uric acid not via CRP is 0.053 (standard error 0.035). This is similar to the total effect of 0.052 (0.032). The indirect effect is −0.001 (0.016). Using the multiple-stage least squares method, again adjusting for age, sex and centre in all the regression stages, the estimates of total, direct and indirect effect are 0.052 (0.033), 0.053 (0.037) and −0.001 (0.017), respectively. Using the structural equation modelling approach, we first standardized the measures of BMI, CRP and uric acid by adjusting for sex, age and centre. This was because there was poor convergence in the SEM algorithm due to the large number of covariates. Estimates of total, direct and indirect effect are 0.052 (0.032), 0.048 (0.034) and 0.004 (0.013), respectively. Similar results were obtained from all three estimation approaches. We conclude that any effect of BMI on uric acid concentrations does not seem to be mediated via CRP levels.

**Table 2.** Coefficients (standard errors) and *P*-values from regression of body mass index (BMI), C-reactive protein (CRP) and uric acid on allele scores for each of the variables in turn. Adjustment is made for sex, age, and centre

Score	BMI	*P*-value	CRP	*P*-value	Uric acid	*P*-value
Allele score for BMI	1.00 (0.12)	<0.001	0.08 (0.03)	0.009	0.05 (0.03)	0.12
Allele score for CRP	−0.83 (0.61)	0.17	1.00 (0.16)	<0.001	−0.01 (0.17)	0.95
Allele score for uric acid	−0.12 (0.21)	0.57	−0.05 (0.05)	0.37	1.00 (0.06)	<0.001

## Discussion

In this paper, we have considered the assessment of the direction of effect between two variables, and the estimation of direct and indirect effects using genetic variants as instrumental variables for the exposure and mediator. The regression-based and SEM methods discussed in this paper give similar estimates, which are consistent in the presence of unmeasured confounding, under the instrumental variable assumptions together with further assumptions on the linearity of effects without interaction terms and on the homogeneity of individual-level effects of the exposure on the mediator and the exposure and mediator on the outcome. A simulation study suggests that random heterogeneity in the effects between the exposure, mediator and outcome does not lead to substantial bias in the estimators of the direct and indirect effects for the wide range of data-generating mechanisms considered; although there was some bias when variability in the individual-level effect parameters was correlated. Additional sensitivity analyses could be performed by proposing different data-generating models; those considered in this paper were chosen as they were thought to be the most likely to occur in applied examples. The methods were illustrated in an applied example, considering the causal relationships between body mass index, C-reactive protein and uric acid.

A theoretical example has been demonstrated with extreme patterns of heterogeneity which would lead to misleading results from a mediation analysis using separate instrumental variables for the exposure and mediator.^35^ In this example, the effects of the exposure on the mediator and of the mediator on the outcome are in different directions for subgroups of the population. Further research to show whether the assumptions of homogeneity of the these effects could be weakened, say to allow heterogeneity in the effects provided they were in the same direction across individuals in the population, would be valuable to add a theoretical result to the simulation findings of this paper.

### Connection to previous literature

The estimation of direct and indirect effects from observational data has received much attention in the recent statistical and epidemiological literature, as well as in numerous other fields.^19^^,^^43–45^ The majority of this literature has been based on the strong and untestable assumption of no unmeasured confounding (of the mediator–outcome relationship as well as the exposure–outcome and exposure–mediator relationships);^13^ extensive work on sensitivity analyses in relation to this assumption has also been published.^46^^,^^47^ Although restrictive in the sense of requiring the assumption of no unmeasured confounding, this literature has succeeded in relaxing many of the other assumptions on which earlier papers on mediation analysis relied, such as no interaction between exposure and mediator, and the linearity of relationships.

**Figure 6.**
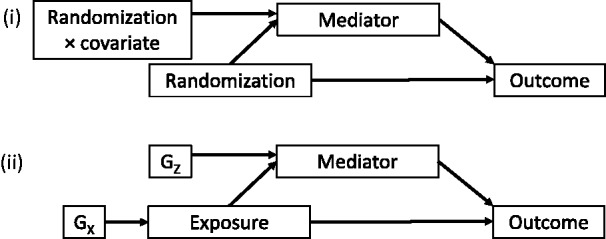
Diagram illustrating mediation scenarios: (i) typically investigated in the context of a randomized trial, (ii) proposed in this paper, with *G_X_* and *G_Z_* representing genetic variants used as instrumental variables. Confounding variables are omitted from the diagram.

In general, there are two approaches for making causal inference from observational data: to assume that there are no unmeasured confounders, or to assume that a variable acts as an IV. The estimation of direct and indirect effects using IVs has been previously addressed in the context of randomized trials (see references 48 and 49 for reviews). In this setting, random assignment is typically used as the exposure, and the interaction between random assignment and a baseline covariate as an IV for the mediator (Figure 6). When using genetic variants as instruments, the association between the IV and the exposure is often weak, and thus using the interaction between this variant and a baseline covariate as an IV for the mediator would typically result in an even weaker IV for the mediator, leading to substantial finite sample bias and imprecision, even if all the IV assumptions were met.^50^ Instead, we have focused on situations in which a different genetic variant can be used as an IV for the mediator. This is a situation similar to that considered by Imai *et al*. in the context of randomized trials^34^ with the first randomization affecting the treatment assigned (analogous to the IV for the exposure) in the whole population, and the second randomization affecting the level of the mediator (analogous to the IV for the mediator) performed in a subsample of participants.

In Mendelian randomization, the genetic variant is not permitted to have an effect on the outcome except via the exposure. Whereas it is possible to consider the direct and indirect effects of a genetic variant on a disease outcome (for example in reference 51), the aim of Mendelian randomization is not to estimate the effect of genes, but the effect of modifiable risk factors on outcomes. Figure 6 illustrates the difference between the use of IVs for mediation analysis in a randomized trial and the Mendelian randomization scenario considered in this paper.

This means that, in the context of randomized trials where the interaction between random assignment and a baseline covariate is used as an IV for the mediator, the assumption of homogeneous effects across individuals is less fundamental than in the Mendelian randomization context considered in this paper. This is because in a randomized trial, only a single randomization ‘experiment’ is performed. A proposal has been put forward to weaken the assumption of homogeneous effects in the context of randomized trials, replacing it with more plausible assumptions that can be assessed by sensitivity analyses.^49^

The use of instrumental variables provides a valuable addition to mediation analysis by relaxing the no unmeasured confounding assumption, but it does so at the cost of reintroducing these stronger assumptions of linearity and no interaction, a trade-off which must carefully be evaluated in any given context.^52^ Interactions between the exposure and mediator could be modelled in a multiple-stage least squares framework,^22^ but were not considered in this paper.

### Violation of the instrumental variable assumptions

Throughout, we have assumed that genetic variants are available which satisfy the IV assumptions for the exposure–mediator and mediator–outcome relationships. This means that an association between the mediator and IVs for the exposure is interpreted as a causal effect of the exposure on the mediator. In practice, it is possible that such associations may reflect pleiotropy (multiple effects of a single gene) rather than mediation. If there are alternative pathways by which variants associated with the exposure may be associated with the mediator, then the assessment of mediation is more problematic. We recommend that investigations into the mediation and the direction of causal effects use genetic variants only where the IV assumptions have a strong biological or scientific basis.

### More complex networks and model selection

The methods and principles used in this paper could be employed to investigate more complex causal networks, either by repeated application of mediation analysis and assessment of the direction of causal effects, or by analysis of a more complex SEM. In many cases, the target of investigation is not the estimation of causal effects, but inference on the underlying set of causal relationships between variables. In a SEM framework, this can be done by testing a series of candidate models. A range of different tests is available in most SEM estimation programs, or standard goodness-of-fit criteria can be used, such as the Akaike information criterion (AIC) or Bayesian information criterion (BIC).^28^ In a Bayesian framework,^27^ additionally the deviance information criterion (DIC)^53^ or the posterior probabilities of models can be compared, for example using Bayes factors.^54^ A similar approach has been suggested to distinguish between causal, reactive and independent models of association using a likelihood-based approach based on the AIC.^55^ Although not all causal models can be distinguished on the basis of observational data, models which have different conditional independence structures result in joint distributions for the variables which can be empirically compared.^56^^,^^57^

Such methods may be useful in large scale ‘omics’ data, such as gene expression data (genomics), methylation data (epigenomics), protein data (proteomics) and transcription data (transcriptomics).^58^ Integration of multiple layers of ‘omics’ data may give us insight into the relations between biomarkers in different layers. Examples of such approaches have been named ‘genetical genomics’ (integration of genetic variants and gene expression data)^59^ and ‘genetical epigenomics’ (integration of genetic variants and epigenetic data).^60^ A practical application of the integration of ‘omics’ data with phenotypic and disease data is documented in the paper of Wan *et al.*^61^—investigating associations between cigarette smoking behaviours and disease outcomes with DNA methylation to search for mechanisms by which an increased risk of smoking-related diseases may persist even after cessation of smoking. Relationships between epigenetic markers, transcription factors and proteins can be affected by confounding and reverse causation in the same way as relationships between phenotypic exposures and outcomes. Although the causal network is generally high-dimensional and unknown, the direction of potential causal relationships between layers of data can often be deduced from external biological knowledge. Relton *et al*.^10^ proposed a similar analytical approach that considered, in this paper under the name ‘two-step epigenetic Mendelian randomization’, using separate genetic variants as instrumental variables for a phenotype (exposure) and an epigenetic marker (mediator), to investigate mediation. A key difficulty here is finding genetic variants specifically associated with the phenotype and with the epigenetic marker if the two variables are closely biologically related.

## Supplementary Data

Supplementary data are available at *IJE* online.

Supplementary Data
