# Supplementary material for: Network Mendelian randomization: using genetic variants as instrumental variables to investigate mediation in causal pathways
Source: Int J Epidemiol. 2014 Aug 22;44(2):484–95. doi: 10.1093/ije/dyu176 (PMC4469795; doi:10.1093/ije/dyu176)
Supplement: Supplementary Data [file supp_44_2_484__index.html]

Network Mendelian randomization: using genetic variants as instrumental variables to investigate mediation in causal pathways — Supplementary Data 

# Network Mendelian randomization: using genetic variants as instrumental variables to investigate mediation in causal pathways

## Supplementary Data

files

**Files in this Data Supplement:**

- Supplementary Data - pdf file
