# Supplementary material for: Network Mendelian randomization: using genetic variants as instrumental variables to investigate mediation in causal pathways
Source: Int J Epidemiol. 2014 Aug 22;44(2):484–95. doi: 10.1093/ije/dyu176 (PMC4469795; doi:10.1093/ije/dyu176)
Supplement: Supplementary Data [file supp_dyu176_dyu176_suppl_data.pdf]

Web Appendix

A.1 Definitions of direct and indirect effects

Indirect and direct effects come in several flavours. We express these using the terminology of the Rubin causal model [1] in terms of potential values of the outcome $Y(x, z)$, representing the outcome which would be observed if X were set (by intervention) to x and Z were set to z, and potential values of the mediator $Z(x)$, the value taken by the mediator if X were set to x. All effects are given on the difference scale; with a binary outcome, effects on a relative risk or odds ratio scale can also be defined, but the decomposition is more complex [2, 3].

A total effect is defined as the effect on the outcome of a change in the exposure from, say, $X = x$ to $X = x+1$. It comprises the effects of the change in the exposure, and the change in the mediator as a result of the change in the exposure:

$$TE(x, x + 1) = Y(x + 1, Z(x + 1)) - Y(x, Z(x)) \tag{1}$$

A controlled direct effect is defined as the effect of a change in the exposure keeping the mediator fixed at a given level, say $Z = z$ [4, 5]. The controlled direct effect may depend on the choice of z:

$$CDE(z; x, x + 1) = Y(x + 1, z) - Y(x, z) \tag{2}$$

A natural direct effect is defined as the effect of a change in the exposure with the mediator fixed at the level it would naturally take if the exposure were fixed at a given level, say $X = x$:

$$NDE(x; x, x + 1) = Y(x + 1, Z(x)) - Y(x, Z(x)) \tag{3}$$

A natural indirect effect is defined as the effect of a change in the mediator from the value it would naturally take if the exposure were unchanged to the level it would take if the exposure were changed. The exposure itself is kept fixed at a given level, say $X = x + 1$:

$$NIE(x + 1; x, x + 1) = Y(x + 1, Z(x + 1)) - Y(x + 1, Z(x)) \tag{4}$$

In the linear case, the natural direct and indirect effects represent a decomposition of the total effect, in that $TE(x, x + 1) = NDE(x; x, x + 1) + NIE(x + 1; x, x + 1)$ (or alternatively $TE(x, x+1) = NDE(x+1; x, x+1)+NIE(x; x, x+1)$). Under the condition:

$$Y(x + 1, z_1) - Y(x, z_1) = Y(x + 1, z_2) - Y(x, z_2) \tag{5}$$

for all values of $Z = z_1, z_2$, and for all individuals, the controlled direct effect is equal to the natural direct effect [4]. The natural direct effect has a clearer intuitive interpretation as a measure of mediation than the controlled direct effect, which can be interpreted even if Z is not a mediator. However, it is not possible to conceive of an experiment which would produce the natural direct effect, as the quantity requires the

outcome if the exposure were set at two different levels (for example, in $NDE(x; x, x+1)$, $Y(x+1, Z(x))$ requires $X = x+1$ for Y, but $X = x$ for Z). This is known as a "cross-world" quantity, as setting the exposure to two different values is only possible in two different worlds [6].

More generally, in a non-parametric context, evaluation of natural direct and indirect effects requires the distribution of $Y(x, Z(x'))$. This can only be evaluated under the assumption that $Y(x, z)$ is independent of $Z(x')$ for $x \neq x'$. This is a cross-world assumption and cannot be empirically verified. Even if the distributions of $Y(x, z)$ and $Z(x)$ can be estimated, for example using instrumental variables, it is not possible to express an estimate of the natural direct or indirect effect without making the cross-world assumption. In contrast, estimation of the controlled direct effect does not require any cross-world assumption, and can be obtained directly at a given value of $X = x$ and $Z = z$ from estimates of the distributions of $Y(x, z)$ and $Z(x)$.

A.2 Impact of interactions on estimates of the direct and indirect effect

To assess the impact of an interaction between X and Z in their effect on Y on estimates of the direct and indirect effects, we perform further simulations. Data were simulated on 5000 individuals indexed by i from the following data-generating model:

$$\begin{aligned}
x_i &= \alpha_G g_{Xi} + u_{1i} + u_{2i} + \epsilon_{Xi} \qquad (6)\\
z_i &= \beta_G g_{Zi} + \beta_{Xi} x_i + u_{1i} + u_{3i} + \epsilon_{Zi}\\
y_i &= \gamma_{Xi} x_i + \gamma_{Zi} z_i + \gamma_{XZi} x_i z_i + u_{2i} + u_{3i} + \epsilon_{Yi}\\
\beta_{Xi} \sim \mathcal{N}(\mu_{\beta_X}, \tau^2), \gamma_{Xi} \sim \mathcal{N}(\mu_{\gamma_X}, \tau^2), \gamma_{Zi} &\sim \mathcal{N}(\mu_{\gamma_Z}, \tau^2)\\
\gamma_{XZi} &\sim \mathcal{N}(\mu_{\gamma_{XZ}}, \psi^2) \text{ independently}\\
u_{1i}, u_{2i}, u_{3i}, \epsilon_{Xi}, \epsilon_{Zi}, \epsilon_{Yi} &\sim \mathcal{N}(0, 1) \text{ independently}\\
g_{Xi}, g_{Zi} &\sim \text{Binomial}(2, 0.3) \text{ independently}
\end{aligned}$$

This model is the same as that considered in the main paper, except that an additional term ($\gamma_{XZi} x_i z_i$) has been added to the data-generating model for Y to allow for an interaction between X and Z. We consider three scenarios for the parameter values ($\mu_{\gamma_{XZ}}, \psi^2$), the mean and variance of γ_{XZi}:

1. $\mu_{\gamma_{XZ}} = 0, \psi^2 = 0.3^2$: interaction is present at an individual level, but absent on average. The average direct and indirect effects of X on Y controlling for Z are $\mu_{\gamma_X} = 1$ and $\mu_{\beta_X} \mu_{\gamma_Z}$, as before. An equivalent model could be achieved by allowing omitting the additional term ($\gamma_{XZi} x_i z_i$) and allowing the γ_{Xi} and γ_{Zi} parameters to be correlated in their distributions.

2. $\mu_{\gamma_{XZ}} = 0.5, \psi^2 = 0^2$: interaction is present, and is homogeneous across individuals.

3. $\mu_{\gamma_{XZ}} = 0.5, \psi^2 = 0.3^2$: interaction is present, and is heterogeneous across individuals.

In both the second and third scenarios, the average direct and indirect effects depend on the interaction between X and Z, and the individual-level direct effects will depend on the value of Z. All other parameters take the same values as in the simulation study in the main paper.

For scenario 1, we present estimates of the direct and indirect effect, and compare these with the theoretical values (Web Table A1). For scenarios 2 and 3, we present estimates of the direct effect only, and compare this with the average direct effect, calculated by adding one to the exposure for each individual in the data-generating model for the outcome but keeping the mediator constant (Web Table A2).

We see that estimates of the direct and indirect effects, which are similarly estimated by regression-based and SEM methods, are not substantially biased by the presence of a zero mean interaction term. However, with non-zero mean interaction,

estimates of the direct effect differ somewhat from the average direct effect. If an interaction between the exposure and mediator is expected, this can be modelled explicitly using the multiple-stage least squares approach [7].

Direct effect ($\mu_{\gamma_X} = 1$)		Regression-based			SEM		
μ_{β_X}	μ_{γ_Z}	$\tau^2 = 0$	0.2^2	0.4^2	$\tau^2 = 0$	0.2^2	0.4^2
1	1	1.01	1.00	1.01	1.00	0.99	1.00
1	−1	1.00	1.00	1.02	0.99	0.99	1.00
−1	1	1.00	1.00	1.00	0.99	0.99	0.99
−1	−1	1.00	1.00	1.01	0.99	0.99	0.99
**Indirect effect ($\mu_{\beta_X}\mu_{\gamma_Z}$)**		**Regression-based**			**SEM**		
μ_{β_X}	μ_{γ_Z}	$\tau^2 = 0$	0.2^2	0.4^2	$\tau^2 = 0$	0.2^2	0.4^2
1	1	0.98	0.98	0.98	0.99	0.99	0.99
1	−1	−1.00	−1.01	−1.01	−0.99	−1.00	−1.00
−1	1	−1.02	−1.01	−1.00	−1.01	−1.00	−0.99
−1	−1	1.00	1.00	1.00	1.01	1.01	1.01

Web Table A1: Mean estimates of the direct and indirect effects of X on Y controlling for Z from regression-based and structural equation model (SEM) methods in simulation study with zero mean interaction between X and Z (Scenario 1)

Scenario 2: Non-zero mean interaction, homogeneous across individuals								
			Regression-based			SEM		
μ_{β_X}	μ_{γ_Z}	Average direct effect	$\tau^2 = 0$	0.2^2	0.4^2	$\tau^2 = 0$	0.2^2	0.4^2
1	1	1.24	1.30	1.30	1.28	1.29	1.30	1.27
1	-1	1.24	1.32	1.30	1.29	1.30	1.29	1.28
-1	1	1.02	0.96	0.96	0.96	0.95	0.95	0.94
-1	-1	1.02	0.97	0.98	0.96	0.96	0.96	0.95

Scenario 3: Non-zero mean interaction, heterogeneous across individuals								
			Regression-based			SEM		
μ_{β_X}	μ_{γ_Z}	Average direct effect	$\tau^2 = 0$	0.2^2	0.4^2	$\tau^2 = 0$	0.2^2	0.4^2
1	1	1.24	1.32	1.26	1.32	1.31	1.25	1.30
1	-1	1.24	1.33	1.30	1.31	1.31	1.29	1.30
-1	1	1.02	0.95	0.97	0.96	0.94	0.95	0.96
-1	-1	1.02	0.96	0.97	0.95	0.95	0.96	0.94

Web Table A2: Mean estimates of the direct effect of X on Y controlling for Z from regression-based and structural equation model (SEM) methods in simulation study with non-zero mean interaction between X and Z (Scenarios 2 and 3)

A.3 Impact of heterogeneity in the genetic effects on estimates of the direct and indirect effect

To assess the impact of heterogeneity in the genetic effects of G_X on X and of G_Z on Z on estimates of the direct and indirect effects, we perform further simulations. Data were simulated on 5000 individuals indexed by i from the following data-generating model:

$$\begin{aligned}
x_i &= \alpha_{Gi} g_{Xi} + u_{1i} + u_{2i} + \epsilon_{Xi} \qquad (7)\\
z_i &= \beta_{Gi} g_{Zi} + \beta_{Xi} x_i + u_{1i} + u_{3i} + \epsilon_{Zi}\\
y_i &= \gamma_{Xi} x_i + \gamma_{Zi} z_i + u_{2i} + u_{3i} + \epsilon_{Yi}\\
\alpha_{Gi} &\sim \mathcal{N}(\mu_{\alpha_G}, 0.1^2), \beta_{Gi} \sim (\mu_{\beta_G}, 0.1^2) \text{ independently}\\
\beta_{Xi} &\sim \mathcal{N}(\mu_{\beta_X}, \tau^2), \gamma_{Xi} \sim \mathcal{N}(\mu_{\gamma_X}, \tau^2), \gamma_{Zi} \sim \mathcal{N}(\mu_{\gamma_Z}, \tau^2) \text{ independently}\\
g_{Xi}, g_{Zi} &\sim \text{Binomial}(2, 0.3) \text{ independently}\\
u_{1i}, u_{2i}, u_{3i}, \epsilon_{Xi}, \epsilon_{Zi}, \epsilon_{Yi} &\sim \mathcal{N}(0, 1) \text{ independently}
\end{aligned}$$

This model is the same as that considered in the main paper, except that the fixed coefficients α_G and β_G are replaced with draws from normal distributions α_{Gi} and β_{Gi} for each individual i. The mean values of these distributions are set at $\mu_{\alpha_G} = 0.3$ and $\mu_{\beta_G} = 0.5$ when $\mu_{\beta_X} = 1$ and $\mu_{\beta_G} = 0.36$ when $\mu_{\beta_X} = -1$. These are the same as the values of α_G and β_G in the original set of simulations. All other parameters take the same values as in the simulation study in the main paper.

Results are given in Web Table A3. No material differences are observed from those in the original simulation study in the main paper. We repeated the simulation except modelling the coefficients α_{Gi} and β_{Gi} by a multivariate normal distribution with correlation 0.4 and -0.4; almost identical results were obtained, with differences between mean values of estimates compatible with chance variation (results not shown).

Direct effect ($\mu_{\gamma_X} = 1$)		Regression-based			SEM		
μ_{β_X}	μ_{γ_Z}	$\tau^2 = 0$	0.2^2	0.4^2	$\tau^2 = 0$	0.2^2	0.4^2
1	1	1.00	1.01	1.01	0.99	0.99	0.99
1	−1	0.99	0.99	1.00	1.00	0.99	0.99
−1	1	1.00	0.99	0.99	1.00	1.00	0.99
−1	−1	1.00	1.00	1.01	0.99	0.99	0.99
**Indirect effect ($\mu_{\beta_X}\mu_{\gamma_Z}$)**		**Regression-based**			**SEM**		
μ_{β_X}	μ_{γ_Z}	$\tau^2 = 0$	0.2^2	0.4^2	$\tau^2 = 0$	0.2^2	0.4^2
1	1	0.98	0.98	0.99	0.99	1.00	0.99
1	−1	−1.00	−0.99	−1.01	−1.00	−1.00	−1.00
−1	1	−1.00	−1.01	−1.02	−1.01	−1.00	−1.01
−1	−1	0.99	1.00	1.00	1.01	1.01	1.01

Web Table A3: Mean estimates of the direct and indirect effects of X on Y controlling for Z from regression-based and structural equation model (SEM) methods in simulation study with heterogeneous genetic effects on X and Z

A.4 Impact of correlations in the causal effect parameters

In the simulations in the main paper, the causal effect parameters β_{Xi}, γ_{Xi}, and γ_{Zi} were allowed to vary between individuals, but they were assumed to vary independently. We perform a further simulation to consider estimates of direct and indirect effects when the parameters vary dependently. Specifically, the vector $(\beta_{Xi}, \gamma_{Xi}, \gamma_{Zi})^T$ for each individual i is drawn from a multivariate normal distribution with mean $(\mu_{\beta_X}, \mu_{\gamma_X}, \mu_{\gamma_Z})$ and variance-covariance matrix consisting of diagonal elements τ^2 and off-diagonal elements $\rho\tau^2$, where ρ is taken to be $+0.4$ and -0.4. This means that the correlation between each pair of β_{Xi}, γ_{Xi}, and γ_{Zi} is ρ. All other aspects of the simulation (including the data-generating model and the parameter values) are taken as in the original set of simulations in the main paper.

Results are given in Web Table A3. No material differences are observed from those in the original simulation study in the main paper for estimates of the indirect effect. Slightly increased estimates of the direct effect are observed with $\rho = +0.4$, and slightly decreased estimates with $\rho = -0.4$, with bias increasing as the heterogeneity parameter τ increases.

	Direct effect ($\mu_{\gamma_X} = 1$)		Regression-based			SEM		
$\rho = +0.4$	μ_{β_X}	μ_{γ_Z}	$\tau^2 = 0$	0.2^2	0.4^2	$\tau^2 = 0$	0.2^2	0.4^2
	1	1	1.01	1.03	1.06	1.00	1.01	1.06
	1	−1	1.02	1.01	1.07	1.01	1.00	1.06
	−1	1	1.01	1.01	1.07	1.00	1.00	1.06
	−1	−1	1.01	1.02	1.07	0.99	1.01	1.07
	Indirect effect ($\mu_{\beta_X}\mu_{\gamma_Z}$)		Regression-based			SEM		
	μ_{β_X}	μ_{γ_Z}	$\tau^2 = 0$	0.2^2	0.4^2	$\tau^2 = 0$	0.2^2	0.4^2
	1	1	0.98	0.98	0.99	1.00	0.99	0.99
	1	−1	−1.01	−1.00	−1.00	−1.00	−0.99	−1.00
	−1	1	−1.01	−1.00	−1.01	−1.01	−0.99	−1.01
	−1	−1	1.00	0.99	1.00	1.02	1.00	1.01
$\rho = -0.4$	Direct effect ($\mu_{\gamma_X} = 1$)		Regression-based			SEM		
	μ_{β_X}	μ_{γ_Z}	$\tau^2 = 0$	0.2^2	0.4^2	$\tau^2 = 0$	0.2^2	0.4^2
	1	1	1.01	1.00	0.92	1.00	0.98	0.92
	1	−1	1.02	0.98	0.94	1.01	0.98	0.93
	−1	1	1.01	0.98	0.94	1.00	0.97	0.94
	−1	−1	1.01	0.99	0.94	0.99	0.98	0.94
	Indirect effect ($\mu_{\beta_X}\mu_{\gamma_Z}$)		Regression-based			SEM		
	μ_{β_X}	μ_{γ_Z}	$\tau^2 = 0$	0.2^2	0.4^2	$\tau^2 = 0$	0.2^2	0.4^2
	1	1	0.98	0.98	0.99	1.00	1.00	1.00
	1	−1	−1.01	−1.00	−1.00	−1.00	−0.99	−0.99
	−1	1	−1.01	−1.00	−1.02	−1.01	−0.99	−1.01
	−1	−1	1.00	0.99	1.00	1.02	1.00	1.01

Web Table A4: Mean estimates of the direct and indirect effects of X on Y controlling for Z from regression-based and structural equation model (SEM) methods in simulation study with correlations ($\rho = \pm 0.4$) in causal effect parameters of X on Z, X on Y, and Z on Y

A.5 Genetic variants and allele scores used in applied example

Genetic variants used as instrumental variables for body mass index (BMI) were: rs2815752, rs1514175, rs11165643, rs543874, rs2867125, rs10182181, rs887912, rs13078807, rs7647305, rs10938397, rs13107325, rs2112347, rs6864049, rs206936, rs987237, rs10968576, rs7127684, rs2030323, rs3817334, rs7138803, rs17109256, rs2241423, rs12444979, rs7359397, rs1421085, rs571312, rs29941, rs2287019, and rs3810291 (29 variants). These were taken from the paper by Speliotes et al. [8] and are located in various regions throughout the human genome; only variants available (or with an available proxy) on the CardioMetabochip (Illumina) were considered, as these were the variants available for the largest proportion of the EPIC-InterAct study population. Weights in the allele score were taken as the coefficients from the Speliotes paper (0.13, 0.07, 0.06, 0.22, 0.31, 0.14, 0.10, 0.10, 0.14, 0.18, 0.19, 0.10, 0.07, 0.06, 0.13, 0.11, 0.06, 0.19, 0.06, 0.12, 0.13, 0.13, 0.17, 0.15, 0.39, 0.23, 0.06, 0.15, 0.09 respectively).

Genetic variants used as instrumental variables for C-reactive protein (CRP) were: rs3093077, rs1205, rs1130864, rs1800947, and rs3091244 (5 variants). These were taken from the paper by Wensley et al. [9], with the addition of rs3091244, which was not considered in the main analysis of this paper. All the variants are located in and around the *CRP* gene region on chromosome 1, which is the coding region for CRP. Weights were taken as the coefficients from the Wensley paper (0.21, 0.18, 0.13, 0.26 respectively), with 0.3 as the weight for rs3091244.

Genetic variants used as instrumental variables for uric acid were: rs4481233 (located in the *SLC2A9* gene region on chromosome 4; this gene encodes a protein which transports uric acid [10]), and rs2231142 (located in the *ABCG2* gene region on chromosome 4; this gene is also involved in uric acid transportation [11]). An unweighted allele score was used (2 variants).
